# Metagenomic analysis and antimicrobial activity of two fermented milk kefir samples

**DOI:** 10.1002/mbo3.1183

**Published:** 2021-05-07

**Authors:** Silvia Tenorio‐Salgado, Hugo G. Castelán‐Sánchez, Sonia Dávila‐Ramos, Alejandro Huerta‐Saquero, Sergio Rodríguez‐Morales, Enrique Merino‐Pérez, Luis Fernando Roa de la Fuente, Sara E. Solis‐Pereira, Ernesto Pérez‐Rueda, Gabriel Lizama‐Uc

**Affiliations:** ^1^ Tecnológico Nacional de México/IT Mérida Mérida México; ^2^ Centro de Investigación en Dinámica Celular Universidad Autónoma del Estado de Morelos Cuernavaca México; ^3^ Centro de Nanociencias y Nanotecnología Ensenada México; ^4^ Unidad de Química‐Sisal Facultad de Química UNAM UMDI‐Sisal Sisal México; ^5^ Departamento de Microbiologia Instituto de Biotecnologıa Universidad Nacional Autonoma de Mexico Cuernavaca México; ^6^ Centro de Investigación de Ciencia y Tecnología Aplicada de Tabasco Universidad Juárez Autónoma de Tabasco Tabasco México; ^7^ Instituto de Investigaciones en Matemáticas Aplicadas y en Sistemas UNAM Unidad Académica Yucatán Mérida México

**Keywords:** fermented milk, kefir, metagenomics, microbiome, prophage sequences, secondary metabolites

## Abstract

In recent years, the fermented milk product kefir has been intensively studied because of its health benefits. Here, we evaluated the microbial consortia of two kefir samples, from Escarcega, Campeche, and Campeche (México). We considered a functional comparison between both samples, including fungal and bacterial inhibition; second, we applied shotgun metagenomics to assess the structure and functional diversity of the communities of microorganisms. These two samples exhibited antagonisms against bacterial and fungal pathogens. Bioactive polyketides and nonribosomal peptides were identified by LC‐HRMS analysis. We also observed a high bacterial diversity and an abundance of *Actinobacteria* in both kefir samples, and a greater abundance of *Saccharomyces* species in kefir of Escarcega than in the Campeche kefir. When the prophage compositions were evaluated, the Campeche sample showed a higher diversity of prophage sequences. In Escarcega, we observed a prevalence of prophage families that infect *Enterobacteria* and *Lactobacillus*. The sequences associated with secondary metabolites, such as plipastatin, fengycin, and bacillaene, and also bacteriocins like helveticin and zoocin, were also found in different proportions, with greater diversity in the Escarcega sample. The analyses described in this work open the opportunity to understand the microbial diversity in kefir samples from two distant localities.

## Graphical abstract

Two kefir samples from Escarcega and Campeche, México, exhibited antagonisms against bacterial and fungal pathogens. We observed a high bacterial diversity and an abundance of Actinobacteria in both kefirs, and a greater abundance of *Saccharomyces* species in kefir from Escarcega. Bioactive polyketides and nonribosomal peptides were identified by liquid chromatography–high‐resolution accurate mass spectrometry (LC‐HRMS) analysis. The sequences of secondary metabolites, such as plipastatin, fengycin, and bacillaene, and bacteriocins (e.g., helveticin and zoocin), were found with greater diversity in the Escarcega sample.

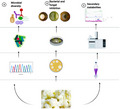

## 1 INTRODUCTION

The use of fermented products has been known to humanity for centuries, and the search for their health benefits has increased in the last decade through research focused on various food products. One of these products of increased interest is kefir. Kefir has been associated with health benefits, such as reduction of blood pressure (Klippel et al., 2016), immunoregulation (De Montijo‐Prieto et al., 2015), and antiallergic, antitumoral, antimicrobial, and anti‐inflammatory effects (Arena et al., 2019; De Montijo‐Prieto et al., 2015; Gao & Zhang, 2019; Hong et al., 2010; Seo et al., 2018; Sharifi et al., 2017) among other health benefits. Kefir is a fermented milk product that is an aggregate of microorganisms, in which lactic acid bacteria, acetic acid bacteria, and yeasts have been reported as predominant (Pogačić et al., 2013; Zhou et al., 2009). However, the microflora and the predominant bacteria in kefir may vary depending on the substrate used in the fermentation process, the method of maintaining the culture, and the geographical, climatic, and cultural conditions, as well as the type of milk used (Marsh et al., 2013; Prado et al., 2015).

There is evidence indicating that microorganisms from a kefir's consortium produce several metabolites, including phosphopeptides, peptides, antibiotics, exopolysaccharides, and bacteriocins, that inhibit the development of degrading microorganisms and pathogens, such as *Salmonella*, *Helicobacter*, *Shigella*, and *Staphylococcus* (Anton et al., 2016; Cleveland et al., 2001; Hong et al., 2010; Lopitz‐Otsoa et al., 2006; Prado et al., 2015). Kefir can also inhibit pathogenic fungi, such as *Candida* *albicans* and *Fusarium* *graminearum* CZ1, among others (Ismaiel et al., 2011; Lopitz‐Otsoa et al., 2006), and it inhibits *Aspergillus* *flavus* formation of spores and production of aflatoxin B1, a toxic compound formed in the crop field or during food storage (Ismaiel et al., 2011). In addition, a high proportion of volatile organic and aromatic compounds in kefir, such as lactic acid, acetic acid, and butyric acid, as well as ethanol, have also been described as important in the inhibition of fungi and bacterial growth (Cais‐Sokolińska et al., 2015; Magalhães et al., 2011).

Therefore, considering the importance of kefir in diverse health and antimicrobial mechanisms, we carried out a systematic study of two kefirs, from Campeche (C_kefir) and Escarcega (E_kefir), by an exhaustive functional analysis describing the antagonistic effects against bacterial and fungal pathogens, complemented with a metabolomic profile compiled from liquid chromatography–high‐resolution mass spectrometry (LC‐HRMS) data to identify probable bioactive compounds involved. Besides, the microorganism and functional diversities were determined for these samples, using a metagenomic shotgun approach. We consider that this analysis opens diverse opportunities to understand the functional role of the microbial consortia, microbial diversity, and their functional profiles within kefir lactic fermented beverage systems and will contribute to knowledge about these environments.

## 2 EXPERIMENTAL PROCEDURES

### 2.1 Kefir sample collection

Two different kefir samples were used for the present study. The first kefir sample, Campeche's kefir (C_kefir), was obtained from Universidad Autónoma de Campeche, Campeche, Mexico (latitude 19.8454, longitude −90.5237; 19° 50′ 43″ north, 90° 31′ 25″ west). The second kefir sample, Escarcega's kefir (E_kefir), was collected from a cattle farm in the municipality of Escarcega, Campeche, Mexico (latitude 18.617, longitude −90.717; 18° 37′ 1″ north, 90° 43′ 1″ west). Both kefir granule samples were kept in the milk of commercial origin, to ensure that the quality of the milk would always be as homogeneous as possible and to diminish significant changes in the microbial consortia during storage.

### 2.2 Titratable acidity

Titratable acidity was determined from fermented milk after 48 hours. To 20 ml of filtered kefir, 3 drops of phenolphthalein were added, after which the mixture was titrated with sodium hydroxide, with the observation of the spent volume until the milk maintained its pink coloration for more than 30 s. It was released three times. The percentages of total organic acids were calculated as follows (according to the Mexican standard NMX‐F‐420‐1982):

$$\mathrm{Acidity}\;\mathrm{titratable}\;g/L\;\left(\mathrm{lactic}\;\mathrm{acid}\right)=\frac{V*N*x}{M}$$

where

*V* = ml 0.1 N NaOH used,

*N* = normality of 0.1 N NaOH, used in sample titration.

*M* = ml kefir used, and

*X* = lactic acid equivalent = 90.

### 2.3 Production and detection of compounds with inhibitory activity

To evaluate the production of antimicrobial metabolites produced by the lactic acid bacteria, consortia were cultured in de Man, Rogosa, and Sharpe (MRS) medium and incubated for 24 h at 25°C. Subsequently, the cultures were centrifuged at 2800 *g* for 20 minutes at 4°C, and the supernatant was filtered through a membrane of 0.22 µm diameter. These extracts were tested against phytopathogenic fungi (*Curvularia* sp., *Fusarium* *equiseti*, and *Colletotrichum* *gloeosporioides*), and against human commensal and pathogenic bacteria (*Escherichia* *coli*, *Salmonella* *typhimurium*, *Bacillus* *subtilis*, and *Staphylococcus* *aureus*), using the method of Kirby‐Bauer for bacteria and radial inhibition percentages for fungi (Bauer et al., 1966).

### 2.4 Antifungal activity

We evaluated the activity of kefir against the growth of three pathogenic fungi, *Curvularia* sp., *F. equiseti*, and *C. gloeosporioides*. Granules of kefir from Campeche and Escarcega were cultured on MRS medium at 25°C for 48 hours with no shaking. After that, the culture was spotted over potato dextrose agar (PDA) culture medium, and PDA plates together with one fungus at a time were incubated for 15 days, with results checked every 5 days. The zones of inhibition (in mm) were determined by measuring the distance between the edges of the fungal mycelia and the bacterial streak. Both kefirs were evaluated in three independent replicates. The kefir antagonistic effect against the fungi was evaluated in three independent replicates and was calculated based on the radial inhibition percentages, according to the following equation.

$$\mathrm{Radial}\;\mathrm{inhibition}\;\left(\%\right)=\left(\frac{Rc‐Ri}{\mathrm{Rc}}\right)*100$$

where *Rc* is the mean value of the fungus radius in the absence of kefir and *Ri* represents the fungus radius in the presence of the consortium.

### 2.5 Antibacterial activity

To evaluate the antibacterial activity against four bacteria, we tested two human commensal bacteria (*E. coli* and *B. subtilis*) and two pathogens (*S. typhimurium* and *S. aureus*). Fresh medium was inoculated from the overnight culture and grew until it reached turbidity of 0.1 optical density (OD) at 600 nm, equivalent to 1.5 × 108 CFU/ml, according to the McFarland index. Subsequently, cultures were diluted to a concentration of 1 × 104 CFU/ml. Next, in 96‐well plates, 100 μl of culture was exposed to 100 μl of the different concentrates of kefir and serial dilutions thereof. All treatments were performed in triplicate. The plates with the treatments were incubated overnight at 37°C and 180 rpm. Finally, 5 μl of each treatment was inoculated on LB agar plates, using a stamper, and incubated for 24 h at 37°C.

### 2.6 Metabolite identification by nontargeted LC‐HRMS analysis

Lyophilized samples were diluted with water of LC‐MS‐grade to obtain 10‐mg/ml working solutions, and then, all the samples were filtered with 0.22 um polytetrafluoroethylene membranes. Samples were injected in an Agilent 1260 LC coupled to an Agilent 6545 hybrid quadrupole time of flight HR‐MS (QToF/HRMS) with a jet stream electrospray ionization source. Samples were analyzed in positive mode; 0.1% formic acid was added to all mobile phases to induce compound ionization. QToF/HRMS detector conditions were capillary voltage of 3500 V; drying gas N_2_, 10 ml/min flow at 300°C; sheath gas at 10 ml/min at 350°C; nozzle voltage 1000 V; fragmentor energy 70 V; and skimmer 65 V. Mass correction was enabled during analysis by injecting standards: 121.0509 *m*/*z* (purine, C_5_H_5 _N_4_) and 922.0098 *m*/*z* [hexakis (1H,1H,3H‐tetrafluoropropoxy)phosphazine, C_18_H_19_O_6_N_3_P_3_F_24_)]. The mass detector was operated in the 2‐GHz extended dynamic range, and the acquisition velocity was 3 spectra/second.

### 2.7 Chromatographic analysis

Sample separation was performed in a Biozen C18 RE XB column (100 × 21 mm, 1.7 mm particle size), with water and acetonitrile as mobile phases A and B, respectively. Both phases were used in a gradient mode with a flow of 0.2 ml/min: 0% B for 3 min, 15% B for 5 min, followed by 35% in 5 min, increased to 100% in 7 min, leaving 100% B for 4 min, returning to 100% A in 4 min, finishing in 30 min of analysis. For the analysis, 5 μl was injected. After separating, peaks were passed to the MS detector.

### 2.8 Bioactive metabolite identification

The identity of compounds in the extracts was determined by searching the monoisotopic accurate mass generated during the analysis in the MetLin commercial database implemented in the Agilent Masshunter program integrated with the PCLD software. MetLin comprises 29,000 exogenous and endogenous natural products from diverse sources, including actinomycetes. Alternatively, an in‐house *Bacillus* and *Gordonia* bioactive metabolite database was built after retrieving data reported in the literature (Farzand et al., 2019; Nagao et al., 2001; Pan et al., 2019; Phister et al., 2004; Xu et al., 2018), using the PCLD program in the Masshunter program (Agilent Technologies). Special care was taken with the sodium and potassium adducts that sometimes form in an MS analysis.

### 2.9 DNA extraction and sequencing

The genomic DNA of C_kefir and E_kefir was extracted using the MoBio Power Soil kit. The samples obtained were sequenced using the Illumina platform (Illumina, 2017). The DNA concentration was determined using a NanoDrop 1000 spectrophotometer (Thermo Scientific). The DNA was sequenced using the Illumina NextSeq 500 platform with the Nextera V2.0 kit (150 bp, 2 × 75 bases) at the Instituto de Biotecnología, Universidad Nacional Autónoma de México.

### 2.10 Taxonomic annotation

The quality of reads was assessed by using FASTQC v0.11.4 software (Andrew, 2010). Subsequently, the reads were trimmed and adapters were removed using Trimmomatic software v.0.38. The reads were assembled using Megahit v 1.1.1–2, with a ‐*k*_min_ = 21, *k*_max_ = 99. The taxonomic annotation was performed using two strategies: In the first one, the assembly contigs were annotated by Kaiju (Menzel et al., 2016); alternatively, a second strategy the Metagenomic Rapid Annotations server using Subsystems Technology (MG‐RAST) was considered.

The diversity index was evaluated using alpha and beta descriptors within the Phyloseq library, and sampling effort was evaluated through the rarefaction curves using a Vegan library implemented in R.

### 2.11 Prophage sequence search in metagenomes

For the identification of prophage, the first approach was through a comparison in BLAST, using a complete genome database of viruses, containing 6000 genomes, with the following parameters: the number of alignments = 20, e‐value = 0.0001, and word size = 11. Subsequently, MEGAN was used to perform the taxonomic classification based on the lowest common ancestor (LCA) and the parameters minimum support = 2, minimum score = 70, top percent = 10. The second strategy that was used was through VIBRANT stand‐alone (Kieft et al., 2020). Briefly, using the hybrid machine learning and similarity of proteins approach to recover the complete virus, the parameters used were contigs with a minimum length of 1000 bp, summary plots on, and function virome off, and the ORF number per scaffold was set to 4 to limit the input to sequences.

## 3 RESULTS AND DISCUSSION

### 3.1 Morphological and physicochemical features of kefir granules

The kefir granules were collected from two locations in the southeast of Mexico, Campeche (C_kefir) and Escarcega (E_kefir). The granules of both kefirs exhibited a similar lobular and irregular shape; C_kefir has granules of 2–4 mm in diameter that are milky white with a firm and viscous texture. In counterpart, granules of E_kefir have a size of 1–2 mm in diameter and a creamy color. These morphological characteristics have been also reported for Argentinean and Tibetan kefirs (Chen et al., 2015; Garrote et al., 2001). At 48 h, both consortia presented a white creamy and carbonated consistency; the pH was 3.7 and 3.6 for C_kefir and E_kefir, respectively, that is, they were not significantly different, as already reported after 48‐h incubation (Garrote et al., 2001).

In addition, the titratable acidity observed in C_kefir was 0.733 g/L, while for E_kefir, it was 0.792 g/L. It is known that organic acids are the major end products of milk fermentation at 48 hours and are associated with a pH decrease, making an acidic environment (Garrote et al., 2001; Sung‐Ho et al., 2013). These organic acids are also associated with the organoleptic and antagonistic properties of kefir (Bengoa et al., 2019). In summary, morphological characteristics and physicochemical properties revealed that pH and titratable acidity were similar between the two kefir samples and were consistent with previous descriptions (Garrote et al., 2001; Hong et al., 2019; Sung‐Ho et al., 2013).

### 3.2 Kefir exhibits an antagonistic effect against fungal pathogens

**FIGURE 1:**
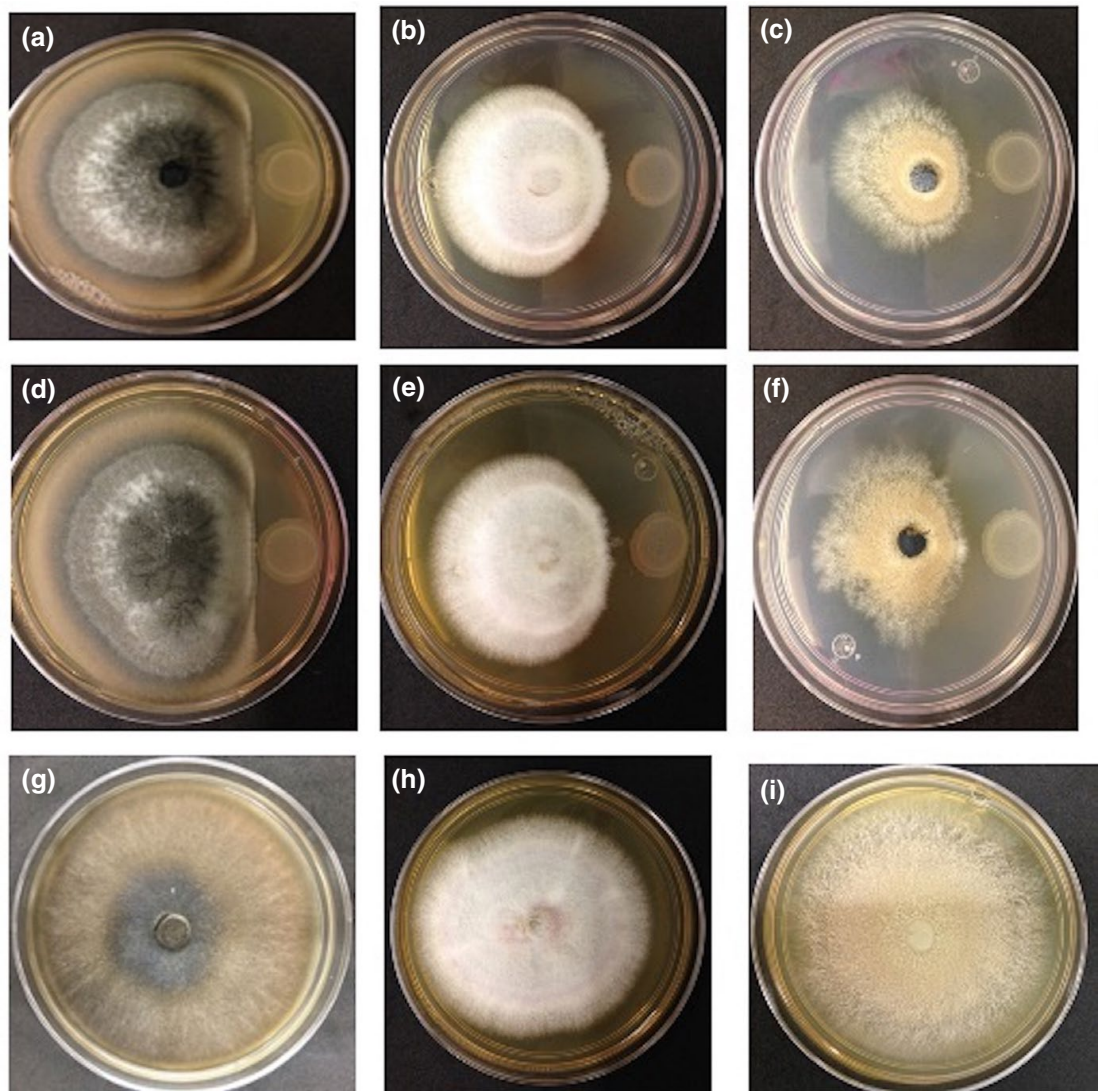
Radial growth inhibition shows the antagonistic effect of the total kefir extracts against three fungi. Columns are as follows: *C*. *gloeosporioides*. (Column 1), *Curvularia* sp. (Column 2), and *F*. *equiseti* (Column 3). In lines are the E_kefir (Line 1), C_kefir (Line 2), and Control, fungi growing in PDA medium with no extract (Line 3). The % of radial inhibition is shown. *n* = 3

It has been described that kefir inhibits pathogenic fungi, such as *C*. *albicans*, *F. graminearum* CZ1, and *A. flavus*. Therefore, to determine whether both Campeche and Escarcega kefirs exhibited antifungal activities, suspensions and cell‐free extracts were evaluated. The first experiment considered a total suspension of both kefirs in a dual‐culture antagonism assay on PDA plates with three phytopathogenic fungi, *Curvularia* sp., *Fusarium* *equiseti*, and *Colletotrichum* *gloeosporioides*. These fungi were selected because they have a wide spectrum of hosts and cause great loss of crops in Mexico. The inhibitory activity was determined based on the limited growth of fungal mycelia in the inhibition zone. In Figure 1, we show that the highest inhibition corresponds to *C. gloeosporioides* with 71% radial inhibition with the E_kefir and 59% radial inhibition with C_kefir. For *Curvularia* sp., the inhibition was 68% with E_kefir and 56% with C_kefir, whereas for *F. equiseti*, it was 50% and 40% with C_kefir and E_kefir, respectively. These results indicate that both kefir suspensions inhibit significantly the three phytopathogenic fungi tested in this assay.

**FIGURE 2:**
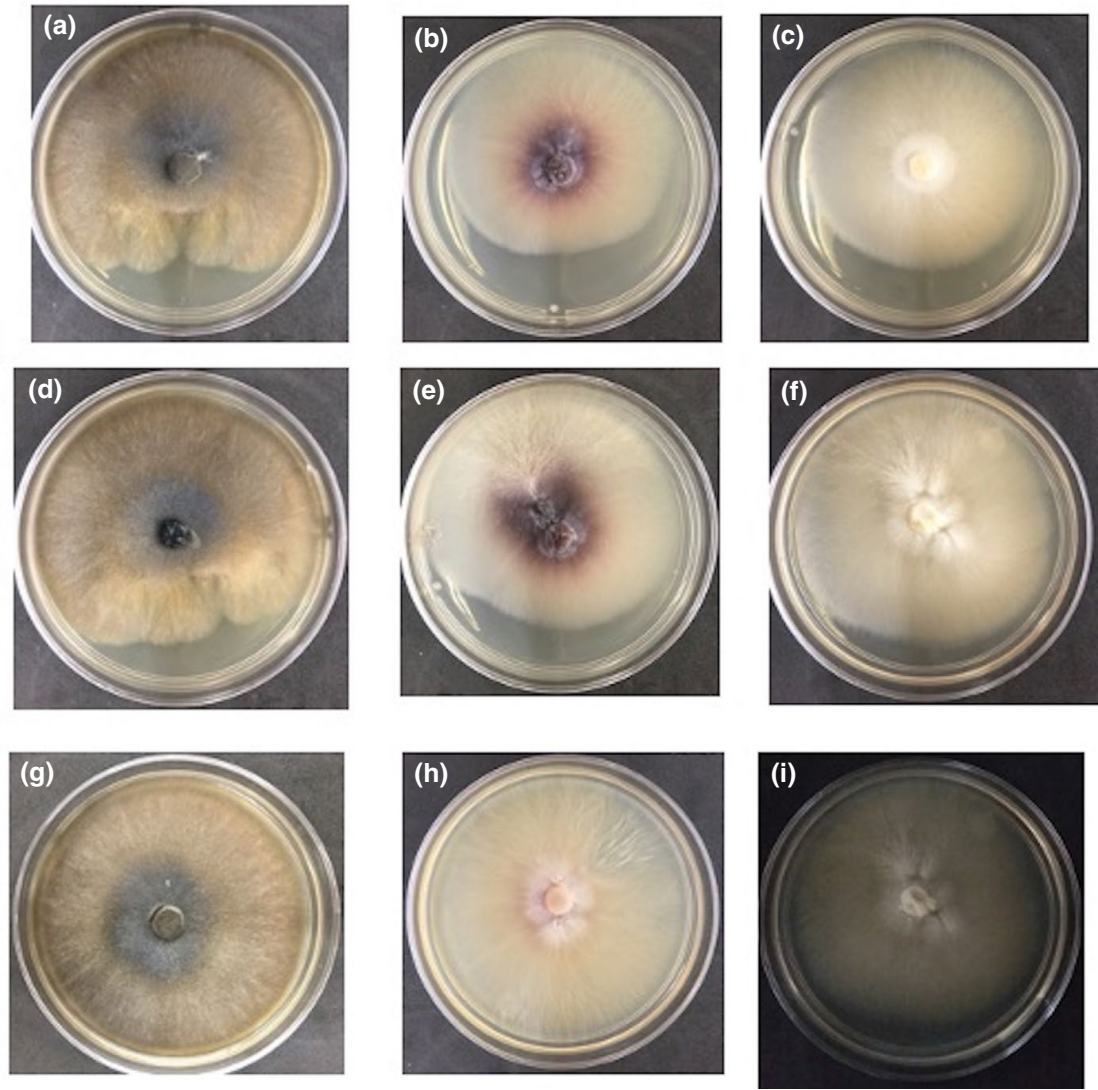
Radial growth inhibition shows the antagonistic effect of the total kefir cell‐free extracts against three fungi. Columns are as follows: *C*. *gloeosporioides*. (Column 1), *Curvularia* sp. (Column 2), and *F*. *equiseti* (Column 3). In lines are the E_kefir (Line 1), C_kefir (Line 2), and Control, fungi growing in PDA medium with no extract (Line 3). The % of radial inhibition is shown. *n* = 3

To determine whether the inhibition observed with the two consortia was due to compounds secreted by the microbial community that comprises the kefir, cell‐free extracts were obtained and antagonistic experiments were carried out. The cell‐free extracts of both consortia inhibited the growth of all fungal organisms evaluated in this work, *Curvularia* sp., *F. equiseti*, and *C. gloeosporioides* (Figure 2), suggesting that these kefir microbial consortia secrete antifungal compounds. The antimicrobial properties of kefir have been mainly associated with the presence of organic acids, such as lactic and acetic acids (Iraporda et al., 2017), making an acidic environment as described above. Indeed, lactic and propionic acids are the main metabolites that inhibit *A. fumigatu*s and *A. nidulans* (Lind et al., 2005). It has also been reported that the antifungal activity of 91 isolates of lactic acid bacteria was attributed to the presence of lactic, acetic, and phenylacetic acids and by a peptide produced by *Lactobacillus* *fermentum* (formally *Limosilactobacillus* *fermentum*) (Gerez et al., 2013; Zheng et al., 2020). Similarly, the antifungal activity can be attributed to the synergistic effect between all the organic acids of the fermentation and by antimicrobial peptides (Arena et al., 2019). In summary, our results suggest that the inhibition of kefir is the result of not only molecules secreted by the microbiota but also the competition for the niche and/or for nutrients, as the inhibition observed with cell‐free extracts was less extensive than the antagonistic effect by the total kefir extracts. In this regard, the main mechanism of inhibition of lactic acid bacteria is by a synergistic effect between the metabolites secreted and the competition for niche and nutrients (Gao & Zhang, 2019; Honoré et al., 2016; Siedler et al., 2019).

### 3.3 Antibacterial activity by cell‐free extracts of kefir

**FIGURE 3:**
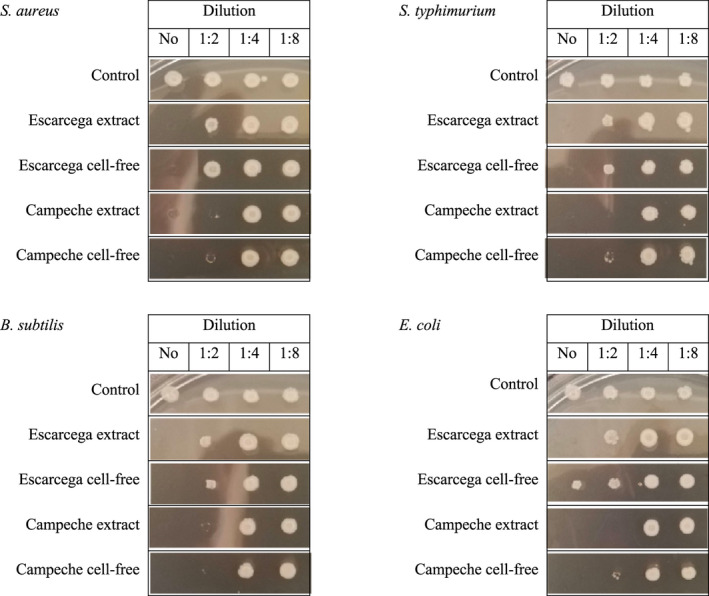
Antimicrobial activity of C_kefir and E_kefir extracts. The assay was realized with *S*. *aureus* *WT*, *E*. *coli* *MG1655*, *S*. *typhimurium* *ATCC* *14028*, and *B*. *subtilis* *ATCC* *23857*. Three dilutions were evaluated, 1:2, 1:4, 1:8, and No dilution

To determine whether the extracts with antifungal activity (described above) also exhibited antibacterial activity, the cell‐free extracts were challenged against two pathogenic bacterial strains, *S. typhimurium* ATCC 14028 and *S. aureus* WT, and two commensal bacterial strains, *E. coli* MG1655 and *B. subtilis* ATCC 23857. From this assay, we found that the C_kefir and E_kefir extracts inhibited the four bacterial strains, at no dilution, dilution of 1:2, and dilution of 1:4 (Figure 3). Also, the E_kefir extracts showed bactericidal activity against the four bacterial strains with no dilution and a dilution of 1:2, considering that no growth of colonies was observed in plates of culture medium seeded in incubation after 24 h. Therefore, C_kefir cell‐free extracts showed increased bactericidal activity against the four bacterial strains, in comparison with the E_kefir extracts, suggesting that C_kefir is more efficient in inhibiting bacterial growth. In this address, antagonistic effects of lactic acid bacteria against *Salmonella*, *Escherichia*, *Staphylococcus*, and *Bacillus* have been reported (Digaitiene et al., 2012; Iraporda et al., 2017; Kim et al., 2016; Nguyen et al., 2015; Silva et al., 2009; Sindi et al., 2020). Indeed, lactic acid bacteria isolated from kefir reduce *Salmonella* infection in epithelial cells *in* *vitro* (Zavala et al., 2016). Hence, the antimicrobial spectrum and potency depend on the type of kefir and the fermentation time, detecting the widest and strongest antimicrobial spectrum between 36 and 48 h of kefir fermentation (Kim et al., 2016). Therefore, the production of some inhibitory compounds, such as bacteriocins, hydrogen peroxide, and organic acids, might be responsible for killing pathogenic microorganisms (Silva et al., 2009). Our results suggest that both cell‐free extracts from C_kefir and E_kefir have antifungal and antibacterial activities, probably related to the production of compounds secreted by the microbiota that conform to both kefirs.

### 3.4 Metabolomic profile by nontargeted LC‐HRMS

**FIGURE A1:**
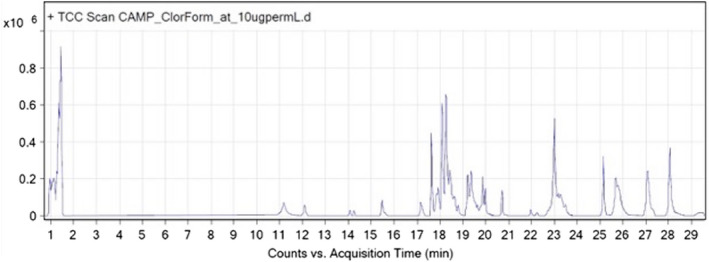
LC‐HRMS profile of the chloroformic extract from Campeche Kefir

**FIGURE A2:**
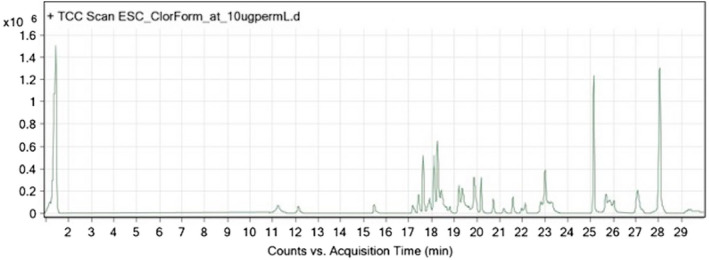
LC‐HRMS profile of the chloroformic extract from Escarcega Kefir

**FIGURE A3:**
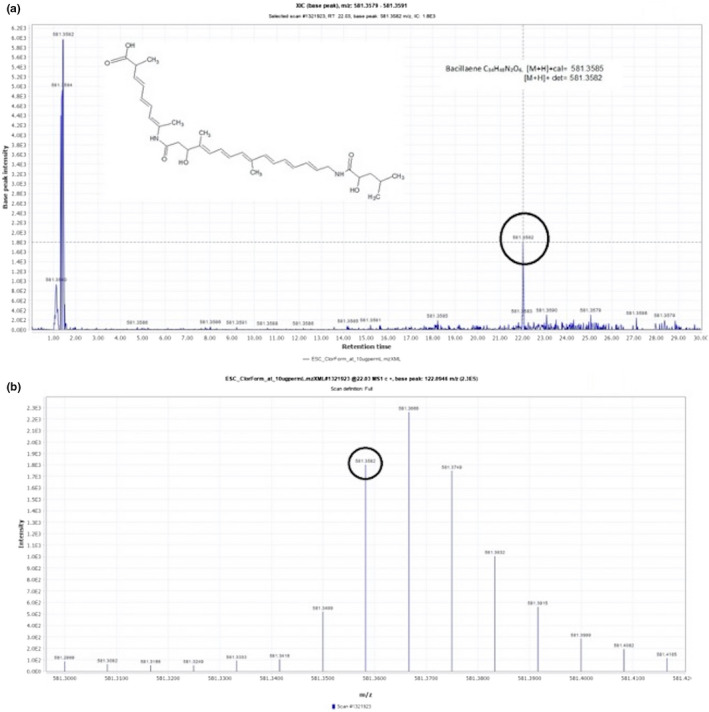
(a) Bacillaene identification from the Chloroformic extract of C_kefir by LC‐HRMS analysis. Protonated accurate monoisotopic mass of bacillaene ([M+H]+calculated: 581.3285 uma) was extracted from the positive ions of the total ion chromatogram (TIC, [M+H]+exp = 581.3285 uma), and the peak was selected according to the retention time obtained in the literature (Farzand et al., 2019). (b) MS spectrum of the accurate monoisotopic protonated mass of bacillaene highlighted by a circle

**FIGURE A4:**
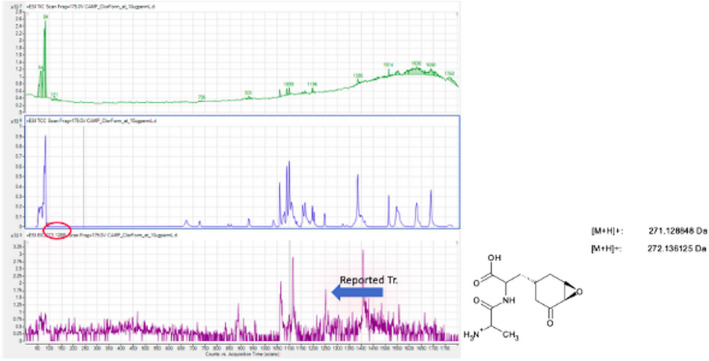
Chromatograms of chloroform extract from Campeche for detection of bacilysin. On the top total ion chromatogram. In the middle, base peak chromatogram (BPC). At the bottom, bacilysin chromatogram

To identify the chemical nature of the compounds, present in the cell‐free extracts of kefir, liquid–liquid organic extraction with chloroform was performed, and metabolomic profiles for both samples were obtained. We used a solvent extraction method because we were interested in amphiphilic molecules, such as bacteriocins produced by lactic acid bacteria. In this regard, hydrophobic regions in antimicrobial molecules are central in their affinity to the lipidic membrane of the cell (Abee et al., 1991; Yusuf, 2013). The analysis showed that E_kefir presents more signals in the chromatogram than the C_kefir consortium. (Figures A1 and A2). Based on a nontarget LC‐HRMS study, we identified 11 different bioactive compounds between the two consortia based on the accurate monoisotopic molecular weight (Table 1 and Figure A3). To do this, we used an in‐house database with biomarkers from different *Bacillus* strains retrieved from the literature (Farzand et al., 2019), because the main group of bacteria in kefir is *Lactobacillus* spp. (Dobson et al., 2011; Gao & Zhang, 2019). Both consortia presented bioactive polyketides (bacillaene, macrolactins, and kammogenin) and nonribosomal peptides (bacilysin and linbacillibactin A). As expected, we identified more bioactive compounds in the E_kefir, such as kammogenin and isomers macrolactins O and T. Of note, our LC‐HRMS analysis could not discriminate between isomers; thus, macrolactin T or O can be present in the extract or both (Figure A4). These compounds have been reported to have antimicrobial activity; for instance, the sphingolipid dehydro‐phytosphingosine, which is present in the membranes of all living organisms, has antibacterial activity and contributes to innate immunity against bacterial infections (Canela et al., 2016; Possemiers et al., 2005), whereas the steroidal sapogenin kammogenin, produced by several plants such as agave, has antimicrobial activity (Guzmán & Contreras, 2018; Jin et al., 2017; Leal‐Díaz et al., 2015; Santos‐Zea et al., 2016). Bacilysin, bacillaene, and macrolactins are polyketides that belong to a large class of structurally diverse natural products that exhibit an extensive set of biological activities, such as antimicrobial activities (Chan et al., 2009; Hill et al., 2017; Park et al., 2017; Schneider et al., 2007). Although the metabolomic profile only differs from two metabolites, kammogenin is lacking in C_kefir and Macrolatin H is presented only in the C_kefir. Indeed, C_kefir showed more concentrated metabolites. This result is consistent with the previous analysis, where compounds produced by kefir exhibit different spectra and activities according to the fermentation time (Kim et al., 2016).

**TABLE 1:** Compounds identified from extracts from E_kefir and C_kefir

Compound	MW	MF	m/z	Monoisotopic mass	Extract C_Kefir	Extract E_Kefir
Dehydro‐phytosphingosine	315.27	C18H37NO3	316.2846	[M + H]+	XX	XX
Dehydro‐phytosphingosine			338.2660	[M + Na]+	XX	XX
Kammogenin	444.28	C27H40O5	467.2762	[M + Na]+		X
Kammogenin			483.2507	[M + K]+		X
Bacilysin	270.28	C12H18N2O5	271.1288	[M + H]+	XX	XX
Bacillaene	580.35	C34H48N2O6	581.3585	[M + H]+	X	X
Bacillaene			603.3391	[M + Na]+	X	
linbacillibactin A	914.82	C40H46N6O19	915.2829	[M + Na]+	X	X
Macrolactin T	418.50	C24H34O6	419.2419	[M + Na]+	XX	XX
Macrolactin U	480.70	C31H44O4	481.3315	[M + Na]+	XX	XX
Macrolactin A	402.24	C24H34O5	403.2401	[M + H]+		XX
Macrolactin O	564.29	C30H44O10	587.2842	[M + Na]+	XX	XX
Macrolactin G	402.24	C24H34O5	425.2304	[M + Na]+		XX
Macrolactin H	376.49	C22H32O5	399.2147	[M + Na]+	XX	

Abbreviations: Accurate molecular monoisotopic ion detected; C_kefir, Chloroformic extract of the consortia from Campeche kefir, E_kefir, Chloroformic extract of the consortia from Escarcega kefir; m/z, accurate monoisotopic mass charge relationship determined for the identity of the metabolite; MF, molecular formula; MW, molecular weight.

Based on these results, open questions remain to be explored: What is the microbial composition of C_ and E_kefirs? Do bacterial consortia produce different compounds associated with their microbial population? Therefore, in the following sections, we describe our main findings associated with a metagenomic analysis to determine the diversity, abundance, and metabolic profiles associated with both kefirs.

### 3.5 Kefir is a consortium integrated by a large proportion of bacteria and Eukarya organisms

To determine the organisms associated with the production of compounds previously described, the microbial and metabolic diversity of two kefir consortia were determined by a metagenomics approach. The results of the sequence assembly revealed a total of 16,166 contigs for C_kefir and 24,138 for E_kefir, containing 19,103,633 and 13,691,381 base pairs (bp). In a posterior step, we were able to assign a taxonomic classification for 14,048 contigs for C_kefir and 20,799 for E_kefir, that is, 86% of the total of contigs.

When the data were analyzed at different taxonomic levels, we found that the diversity of metagenomes at the domain level showed that the E_kefir had 72.8% of sequences assigned to Bacteria, followed by Eukarya (26.8%), while in the C_kefir, 96.72% corresponded to Bacteria and 3% to Eukarya. Therefore, the different compositions at a domain level could influence the production and chemical nature of metabolites.

**FIGURE 4:**
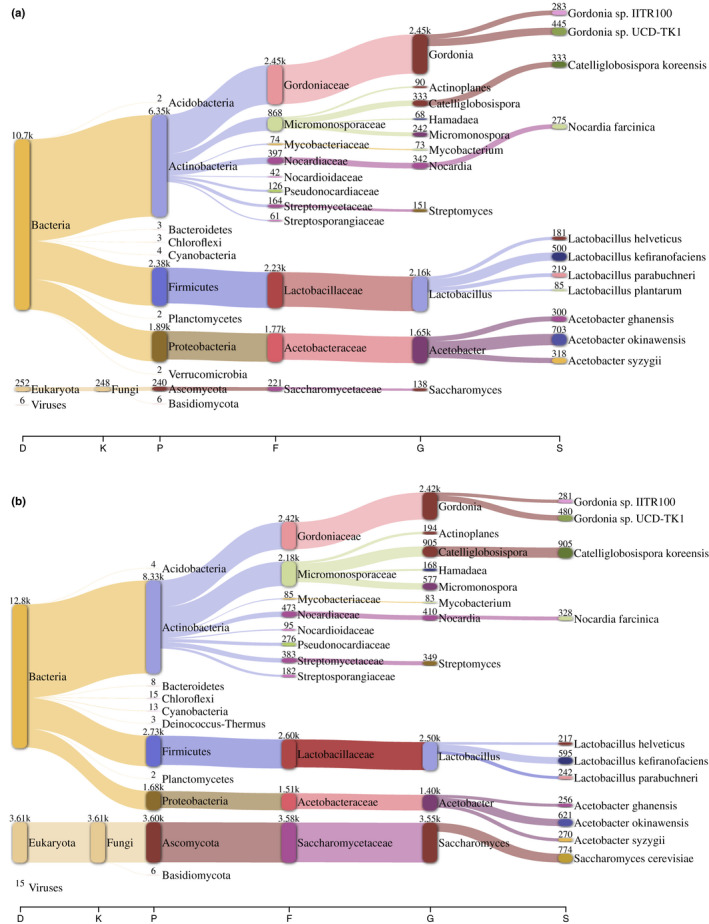
Taxonomic profile in kefir metagenomes. (a) Campeche. (b) Escarcega. On the x‐axis are the taxonomic levels: D, domain; P, phylum; C, class; O, order; F, family; G, genus; S, species. Numbers correspond to the assigned contigs. *Lactobacillus* *parabuchneri* *(formally* *Lentilactobacillus* *parabuchneri)*, *and* *L*. *plantarum* *(formally* *Lactiplantibacillus* *plantarum*)

At the phylum level, we found that Actinobacteria (51.72%), Proteobacteria (23%), and Firmicutes (21.5%) of Bacteria and Ascomycota (3%) of Eukarya were predominant in C_kefir, while in E_kefir, Actinobacteria (45.5%), Firmicutes (14.28%), and Proteobacteria (11.67%) of Bacteria and *Ascomycota* of Eukarya (27.6%) were predominant (Figure 4). This is the first report where Actinobacteria have been detected as the most abundant in kefir, and our findings contrast with previous reports identifying this phylum in small proportions in kefir from Ireland and Italy (Dobson et al., 2011; Marsh et al., 2013).

The presence of Bacteria and Eukarya at different proportions suggests that their contribution could influence the production of more bioactive compounds in the E_kefir and C_kefir. For instance, as reported in water kefir, the interaction in coculture between *L. kefiranofaciens* and *S. cerevisiae* enhances the production of kefiran a polysaccharide with antimicrobial activity (Cheirsilp et al., 2003).

When the metagenomes were analyzed at the genus level, we found 15 different genera in both consortia that accounted for 78% of the sequences found for C_kefir and 86% for E_kefir; the predominant were *Lactobacillus* and *Acetobacter* (Firmicutes), *Gordonia* and *Micromonospora* (Actinobacteria), and *Saccharomyces* (Ascomycota). Figure 4. *Lactobacillus* was the most abundant Firmicutes in both kefirs, 18% for E_kefir and 28% for C_kefir, similar to previous reports (Nalbantoglu et al., 2014; Zalewska et al., 2018), whereas *Gordonia* was the most abundant Actinobacteria in both consortia. Finally, *Saccharomyces* was the most abundant Ascomycota of E_kefir, accounting for 30% of the fungal assignments. In contrast, in C_kefir *Saccharomyces* accounted for 3% of eukaryotes. This result contrasts with previous reports, describing a relative abundance of 0.5% for *Saccharomyces* in this fermented milk (Marsh et al., 2013).

**FIGURE A5:**
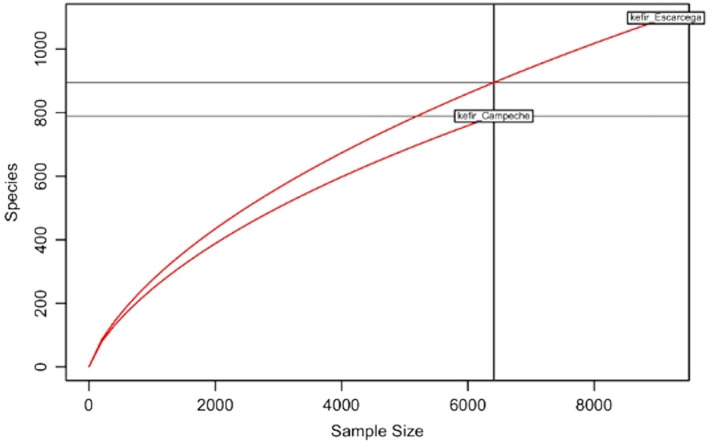
Rarefaction curves based on level species diversity

The indices of richness and evenness were calculated, and the results indicate that the diversity of E_kefir is much greater than the diversity of C_kefir (Table A1). Also, a similar trend can be observed in the rarefaction curve (Figure A5), where E_kefir was close to reaching a horizontal asymptote, compared to C_kefir. These results indicate that E_kefir in general has a more diverse consortium than C_kefir.

### 3.6 Most abundant species in Campeche and Escarcega kefirs

The most abundant bacterial species in C_kefir were *Acetobacter* *okinawensis* (10.9%), *L. kefiranofaciens* (7.8%), *Gordonia* sp. UCD‐TK1 (6.9%), *Catelliglobosispora* *koreensis* (5.1%), *Acetobacter* *syzygii* (4.9%), *Acetobacter* *ghanensis* (4.6%), *Gordonia* sp. IITR100 (4.4%), *Nocardia* *farcinica* (4.2%), *Lactobacillus* *parabuchneri* *(formally* *Lentilactobacillus* *parabuchneri)* (3.4%), *L. helveticus* (2.8%), *L. plantarum* *(formally* *Lactiplantibacillus* *plantarum)* (1.3%), and *L. kisonensis* (*formally* *Lentilactobacillus* *kisonensis*) (1.2%) (Zheng et al., 2020). Regarding E_kefir, the most abundant species found were *Catelliglobosispora* *koreensis* (9.9%), *Acetobacter* *okinawensis* (6.7%), *L. kefiranofaciens* (6.5%), *Gordonia* sp. UCD‐TK1 (5.2%), *Nocardia* *farcinica* (3.5%), *Gordonia* sp. IITR100 (3%), *L. parabuchneri* (2.6%), *L. helveticus* (2.3%), *L. plantarum* (0.97%), and *L. kisonensis* (0.90%). Concerning the Eukarya, we found that *S. cerevisiae* was more abundant in E_kefir (8.4%) than in C_kefir (0.92%). We detected differences in C_kefir and E_kefir with regard to microorganisms present in both consortia, with the major differences observed being *A. okinawensis*, *Lactobacillus* *kefiranofaciens*, and *Gordonia* sp UCD‐TK1, which were more abundant in C_kefir than E_kefir; however, in E_kefir, *C. koreensis* and *S*. *cerevisiae* were more abundant. Likewise, we detected, in both consortia, species that have been previously reported in different kefirs, such as *L*. *kefiranofaciens*, *L*. *helveticus*, *L*. *plantarum*, and *S*. *cerevisiae* among others (Garofalo et al., 2015; Marsh et al., 2013; Sindi et al., 2020). Nevertheless, we found species not previously reported in kefir, such as *Gordonia* sp. UCD‐TK1 and *Catelliglobosispora* *koreensis*. These variabilities in the populations could be associated with the production of different compounds.

### 3.7 Prophage diversity in kefir metagenomes

**FIGURE 5:**
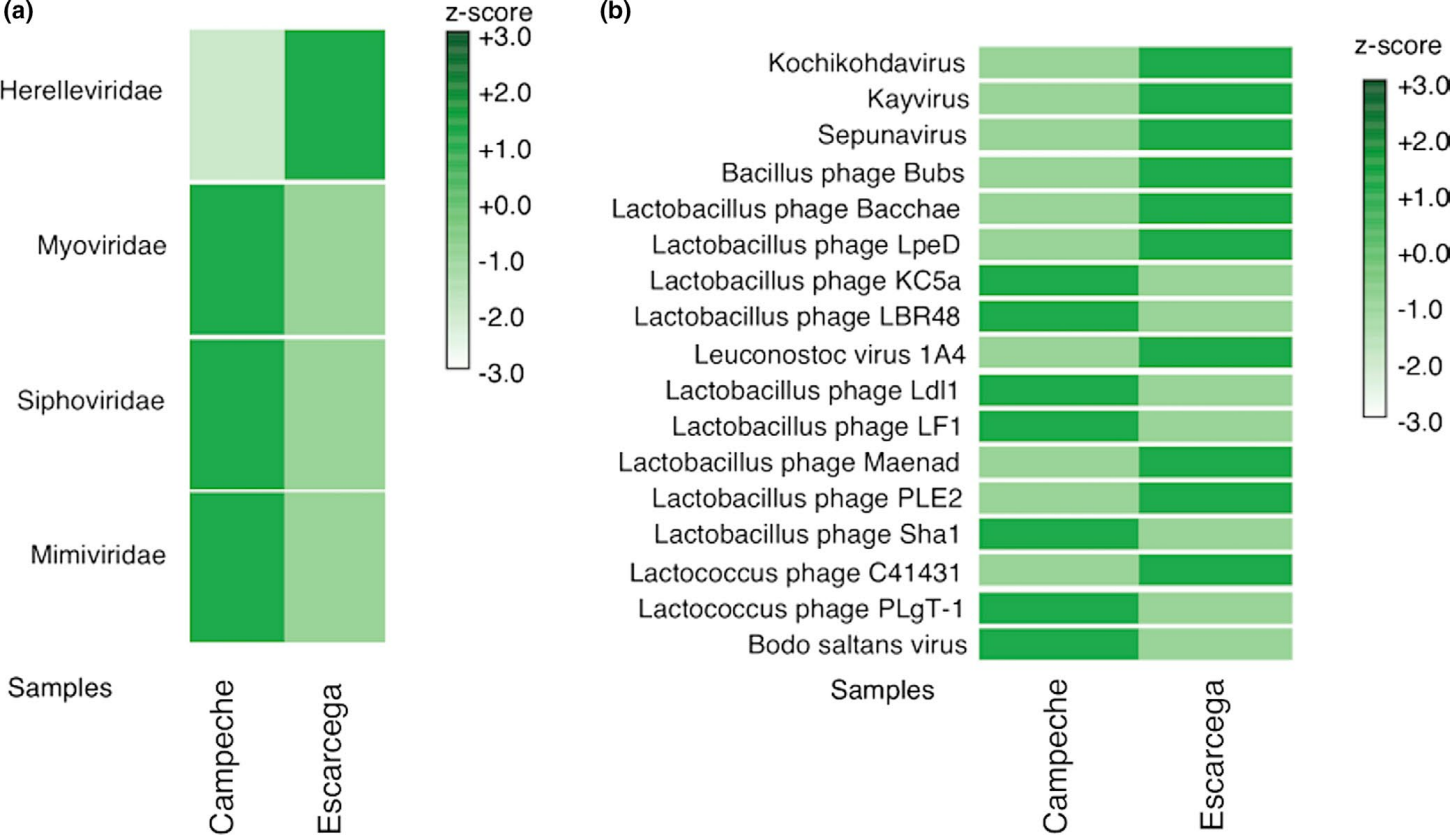
Heatmap of the taxonomic classification of recovered bacteriophage contigs. (a) Family, (b) Species

Bacteriophages play a pivotal role in microbial abundance and metabolism, due to their ability to regulate the competitive relationships among different microorganisms (Mills et al., 2013). To determine the diversity of prophages, we retrieved those prophage sequences from the metagenomic DNA described above. From this analysis, we found in C_kefir 0.19% of the sequences corresponded to prophage sequences, versus 0.25% in E_kefir. According to our results, we found that C_kefir showed a greater diversity of prophages than did E_kefir, and we observed a prevalence of families that infect *Enterobacteria* and *Lactobacillus*, such as *Siphoviridae*, *Myoviridae*, *Microviridae*, *Podoviridae*, and *Herelleviridae*. In particular, the *Lactobacillus* phage Ldl1 and *Lactobacillus* phage Sha1, members of the *Siphoviridae* family, infect bacteria of the *Lactobacillus* genus identified in both kefirs (Mihara et al., 2016; Figure 5). This result correlates with the different proportions of bacteria associated with E_kefir, where there is a predominance of Bacteria and Fungi, versus C_kefir, in particular *Actinobacteria* and Firmicutes, and *Saccharomyces*. Also, it agrees with two *L. plantarum* bacteriophages (Siphoviridae family) having been isolated from Argentinian Kefir grains (De Antoni et al., 2010).

### 3.8 Prediction of secondary metabolites produced by C_kefir and E_kefir

To identify probable genes encoding the biosynthetic pathway for the production of secondary metabolites in the metagenomic sequences of C_ and E_kefirs, the program antiSMASH (Blin et al., 2019) was used. In brief, antiSMASH uses a collection of profiles to predict clusters of genes associated with secondary metabolite biosynthesis pathways. Based on this approach, we identified 18 putative biosynthetic gene clusters in C_kefir and 40 in E_kefir that are responsible for the production of secondary metabolites. These clusters of genes were identified as associated with the production of bacteriocins, polyketides (PKs), and nonribosomal peptides (NRPs), active against a wide range of microorganisms including bacteria, protozoa, yeast fungi, prophages, and even tumor cells, in both kefir samples. In this context, in C_kefir we found 14 out of 18 regions associated with the production of NRPs. These regions were identified with a coverage of 44.5 to 100% (Table A2). These NRPs are described as siderophores (bacillibactin and staphyloferrin A); antibacterial and antifungal compounds (arylomycin, fusaricidin B, fengycin, and friulimicin A, among others), and anticancer compounds (telomestatin), among other activities. Also, we predicted 2 bacteriocins (ecumicin and catenulipeptin) at three different regions, with a coverage of 29%, 67.6%, and 99.6% and E‐values of 3.40E‐06, 8.40E‐46, and 1.30E‐185, respectively, with antibacterial and antibiotic activities. Finally, 2 PKs with a coverage of 93.3% (E‐value = 5.90E‐71) and 96.4% (E‐value = 1.30E‐60), lagriamide and napyradiomycin, associated with antifungal and antimicrobial activities, were also predicted. All these compounds are related to Actinobacteria and Bacillales.

In contrast, in the E_kefir samples, we found 40 regions involved in secondary metabolite biosynthesis pathways, according to the antiSMASH program. From these, 32 out of 40 regions are predicted as NRPs; 2 were predicted as PKs and 4 as bacteriocins (Table A3). From the predicted NRPs, 18 were identified as having probable antibiotic effects (coverage of 23.7%–100%), such as nogabecin, plipastatin, daptomycin, macrotermycins, griseoviridin, vancomycin, and virginiamycin, among others, mainly associated with Actinobacteria (*Streptomyces*) and Bacillales (*Bacillus* and *Paenibacillus*). Indeed, this finding correlates with the fact that E_kefir has a greater proportion of Saccharomycetes than C_kefir. *S. cerevisiae* has been shown to adjust its metabolism to secrete various metabolites, especially amino acids, which allow the survival of lactic acid bacteria (Ponomarova et al., 2017), and amino acids are the main components of NRP and PK scaffolds.

On the other hand, there is a correlation between results observed by LC‐HRMS and antiSMASH. We detected plipastatin in C_kefir extracts by antiSMASH and by LC‐HRMS. Also, we detected in E_kefir extracts difficidin, bacillaene, and plipastatin by LC‐HRMS and antiSMASH. There is a correlation between the highest antimicrobial activity with E_kefir extracts compared with C‐Kefir extracts, agreeing with our results for inhibition, suggesting that E_kefir produces more bioactive secondary metabolites than C_kefir.

The presence of secondary metabolites could explain the antifungal and antibacterial activities of the extracts of both consortia. In this regard, the second group of compounds was predicted, the bacteriocins. Based on an analysis using the BACTIBASE server (Hammami et al., 2010), we found 9 bacteriocins in Campeche and 10 associated with Escarcega (Table A4 and Table A5, respectively). From these, five bacteriocins classified as zoocin A were predicted in Campeche and seven in Escarcega. Zoocin A has been described as a penicillin‐binding protein and presumably is a D‐alanyl endopeptidase, identified in several *Streptococcus* species (Heath et al., 2004).

## 4 CONCLUSIONS

In this work, we studied two kefir samples, from Escarcega and Campeche (México), by two approaches. The first approach was a functional comparison between both samples, including fungal and bacterial inhibition; the second approach used a metagenomic shotgun methodology to assess the structures and functional diversity of the communities of microorganisms. Based on these approaches, we found that these two samples exhibited antagonisms against bacterial and fungal pathogens. Bioactive polyketides (bacillaene, macrolactins, and kammogenin) and nonribosomal peptides (bacilysin, bacillibactin A) were identified by LC‐HRMS analysis. In addition, we observed high bacterial diversity, an abundance of Actinobacteria, and a differential proportion of Ascomycota organisms and prophages. The analyses described in this work provide the opportunity to understand the microbial diversity in kefir samples from two distant localities.

## 5 ETHICS STATEMENT

None required.

## CONFLICT OF INTEREST

None declared.

## AUTHOR CONTRIBUTIONS

**Silvia Tenorio‐Salgado:** Conceptualization (equal); Formal analysis (equal); Investigation (equal); Methodology (equal); Writing‐original draft (equal). **Hugo Castelán‐Sánchez:** Methodology (equal). **SONIA DAVILA:** Writing‐review & editing (supporting). **Alejandro Huerta‐Saquero:** Methodology (equal). **Sergio Rodriguez:** Methodology (equal). **Enrique Merino:** Resources (supporting). **Fernando Roa de la Fuente:** Methodology (supporting). **Sara Elena Solis:** Writing‐review & editing (supporting). **Ernesto Perez‐Rueda:** Methodology (supporting); Resources (supporting); Writing‐review & editing (supporting). **GABRIEL LIZAMA:** Conceptualization (supporting); Investigation (supporting).

## Data Availability

The metagenomes are available in the Sequence Read Archive from NCBI under the BioProject PRJNA704713: https://www.ncbi.nlm.nih.gov/bioproject/PRJNA704713

## 1

**TABLE A1:** Indexes of diversity

Sample	Observed	Chao1	se.chao1	Shannon	Simpson
kefir_Campeche	789	1715.25	126.73	4.46	0.96
kefir_Escarcega	1098	2191.81	124.79	4.67	0.96

**TABLE A2:** Campeche kefir antiSMASH detector clusters of genes associated with secondary metabolite biosynthesis pathways

Region	Type	From	To	Most similar known cluster	% ID	% Coverage	BLAST Score	E‐value	Species	Activity
Region 37.1	lanthipeptide	1	12,117	Catenulipeptin	41	67.6	182	8.40E−46	*Catenulispora acidiphila*	Antibiotic
Region 106.1	NRPS	1	1739	Friulimicin A	50	100.2	496	6.80E−140	*Actinoplanes friuliensis*	Antibiotic
Region 298.1	NRPS‐like	1	2306	Telomycin	42	64.8	237	6.20E−62	*Streptomyces canus*	Antibacterial
Region 399.1	NRPS‐like	1	1180	Fengycin	42	52.6	133	1.00E−30	*Bacillus velezensis*	Antifungal
Region 658.1	NRPS‐like	1	1979	Himastatin	45	80	379	8.10E−105	*Streptomyces himastatinicus*	Antibiotic
Region 823.1	NRPS	1	1429	Plipastatin	45	52.5	208	2.30E−53	*Bacillus subtilis*	Antifungal
Region 897.1	NRPS	1	1638	Bacillibactin	53	80.9	508	1.20E−143	*Bacillus subtilis subsp*	Siderophore
Region 970.1	T3PKS	127,516	156,217	Napyradiomycin	41	93.3	266	5.90E−71	*Streptomyces aculeolatus*	Antimicrobial activity
Region 983.1	arylpolyene	1	5733	Fusaricidin B	41	64.2	163	8.70E−40	*Paenibacillus polymyxa*	Antibacterial
Region 990.1	lanthipeptide	1	7962	Ecumicin	26	29	52	3.40E−06	*Nonomuraea*	Antibacterial
Region 1029.1	NRPS	1	2862	Arylomycin	45	67.4	448	3.50E−125	*Streptomyces filamentosus*	Antibacterial
Region 1030.1	NRPS	1	2207	Rhizopodin	45	44.5	228	3.40E−59	*Stigmatella aurantiaca*	Antibacterial
Region 1161.1	NRPS	1	4688	Ecumicin	35	99.6	649	1.30E−185	*Nonomuraea sp*	Anti‐mycobacterial
Region 1291.1	siderophore	1	1013	Staphyloferrin A	25	84	854	3.20E−07	Catelliglobosispora koreensis	Antimicrobial activity
Region 1390.1	terpene	1	1460	Lagriamide	43	96.4	232	1.30E−60	Acetobacter okinawensis	Antifungal
Region 1463.1	NRPS	1	2423	Daptomycin	45	101.7	568	2.00E−161	*Streptomyces filamentosus*	Bactericidal
Region 1469.1	NRPS	1	2784	Stenothricin	46	101.7	658	2.10E−188	Nocardia	Antimicrobial activity
Region 1813.1	NRPS	1	2217	Balhimycin	38	99.3	411	3.70E−114	Gordonia alkanivorans	Antibiotic

**TABLE A3:** Escarcega kefir antiSMASH detector clusters of genes associated with secondary metabolite biosynthesis pathways

Region	Type	From	To	Most similar known cluster	% ID	% Coverage	BLAST Score	E‐value	Species	Activity
Region 96.1	NRPS	1	1896	Ristomycin A	48	42.5	201	3.80E−51	*Amycolatopsis*	Antibiotic
Region 143.1	lanthipeptide	12,681	24,421	Ecumicin	26	29	52	3.40E−06	*Nonomuraea sp*	Antibiotic
Region 285.1	NRPS	1	3741	Glycopeptidolipid	48	45.6	228	2.90E−59	*Gordonia*	Immunomodulator
Region 314.1	NRPS‐like	1	4802	Colabomycin E	34	42.9	121	5.20E−27	Streptomyces aureus	Anti‐inflammatory
Region 383.1	T1PKS	1	7964	Difficidin	34	23.8	93	1.50E−18	*Bacillus velezensis*	Antibacterial
Region 410.1	NRPS‐like	1	1429	Simocyclinone	47	100.8	363	5.70E−100	*Streptomyces antibioticus*	Antibiotico
Region 529.1	NRPS	1	5784	Cacibiocin B	45	23.7	353	2.40E−96	*Catenulispora acidiphila*	Antibacterial
Region 680.1	NRPS	1	2928	Daptomycin	46	99.8	700	3.10E−201	*Streptomyces filamentosus*	Antibiotic
Region 713.1	NRPS	1	2793	Atratumycin	45	101.6	664	2.30E−190	*Streptomyces atratus*	Antibiotic
Region 729.1	LAP	1	12,396	Mutacin	26	95.9	81	6.30E−15	*Lactobacillus helveticus*	Bacteriocine
Region 764.1	NRPS	1	2251	Acyldepsipeptide	47	95.1	570	4.80E−162	*Gordonia*	Antibiotic
Region 778.1	NRPS	1	5341	Ristomycin A	40	44.5	362	3.80E−99	*Amycolatopsis*	Antibiotic
Region 808.1	NRPS	1	4140	Chivosazol	42	42.9	395	3.90E−109	*Gordonia*	Antibiotic
Region 816.1	NRPS	1	5023	Atratumycin	43	77.8	921	1.90E−267	*Streptomyces atratus*	Antimycobacterium
Region 963.1	lanthipeptide	1	12,117	Lanthipeptide	26	48.8	113	1.60E−24	*Lactobacillus kefiranofaciens*	Antimicrobial
Region 1117.1	NRPS	1	2353	Bacitracin	43	97.1	434	3.30E−121	*Bacillus licheniformis*	Antibiotic
Region 1232.1	arylpolyene	1	4609	Bacillaene	41	64.2	163	8.70E−40	*Bacillus amyloliquefaciens*	Antibiotic
Region 1307.1	NRPS	1	1205	Lactonamycin	47	95.3	306	6.90E−83	*Nocardia farcinica*	Antibiotic
Region 1440.1	NRPS	1	3328	Nogabecin	36	65.7	190	7.20E−48	*Amycolatopsis*	Antibiotic
Region 1630.1	NRPS	1	1849	Paenibacterin	42	100.7	378	2.90E−104	*Paenibacillus*	Antibiotic
Region 1695.1	NRPS	1	3045	Plipastatin	46	29.2	230	1.60E−59	*Bacillus*	Antibiotic
Region 1772.1	NRPS	1	1585	Daptomycin	35	45.3	94	3.90E−19	*Gordonia*	Antibiotic
Region 1802.1	NRPS‐like	1	1349	Kistamicin	40	54.6	129	9.90E−30	*Nonomuraea*	Antibiotic
Region 1896.1	NRPS‐like	1	1923	Atratumycin	45	97.3	433	4.60E−121	*Streptomyces atratus*	Antibiotic
Region 1933.1	NRPS	1	2875	Macrotermycins	41	82.6	477	5.40E−134	*Amycolatopsis*	Antibiotic
Region 1951.1	NRPS	1	2415	Glycinocin A	37	107.1	362	2.10E−99	*Streptomyces viridochromogenes*	Antibiotic/Ca+ dependent
Region 2148.1	bacteriocin	1	1908	Linocin_M18	50	88.4	248	1.10E−65	*Gordonia*	Bacteriocine
Region 2293.1	NRPS‐like,siderophore	1	3146	Desferrioxamine E	49	90.9	294	2.20E−79	*Streptomyces*	Siderophore
Region 2299.1	NRPS	1	4900	Griseoviridin	44	100.4	1059	0.00E+00	*Streptomyces*	Antibiotic
Region 2319.1	T3PKS	127,514	156,306	Daptomycin	40	87.3	201	2.00E−51	*Streptomyces*	Antibiotic
Region 2355.1	NRPS	1	2034	Vancomycin	48	28.5	160	7.90E−39	*Amycolatopsis*	Antibiotic
Region 2376.1	NRPS	1	2312	Virginiamycin	41	55.2	272	1.60E−72	*Streptomyces virginiae*	Antibiotic
Region 2390.1	NRPS‐like	1	1160	Kutzneride	50	69.4	218	1.40E−56	*Kutzeniera*	Antifungal
Region 2529.1	NRPS‐like	1	1778	Bleomycin	43	39.9	128	2.90E−29	*Streptomyces*	Antibiotic
Region 2570.1	NRPS	1	3082	Fuscachelin A	46	42.1	306	1.40E−82	*Thermobifida*	Siderophore
Region 2670.1	NRPS‐like	1	1412	Feglymycin	42	74.7	253	6.20E−67	*Streptomyces*	Antibiotic/AntiHIV
Region 2803.1	ectoine	1	3798	Ectoine	57	90.2	158	1.10E−38	*Streptomyces*	Osmolyte
Region 2938.1	NRPS	1	4385	Sarpeptin	39	67.8	525	2.60E−148	*Streptomyces*	Antibiotic
Region 2989.1	NRPS	1	4812	Antimycin	42	37.2	179	2.10E−44	*Streptomyces*	Anticancer
Region 3100.1	NRPS‐like	1	4674	Ecumicin	39	77.9	342	1.90E−93	*Nonomuraea*	Antibiotic

**TABLE A4:** Bacteriocins detected in Campeche metagenome

Query	Subject acc.ver	Taxonomic Group	Bacteriocin detected	% identity	e‐value
BAC133	k95_7969_1_820_−	Lactobacillales	Class III. Enterolisine A	32.6	1.11E−05
BAC133	k95_6496_1_638_+	Lactobacillales	Class III. Enterolisine A	36.2	1.70E−05
BAC134	k95_11182_1_615_+	Lactobacillus helveticus	Clase II. Helveticina J,	47.1	3.87E−46
BAC137	k95_2274_231_402_−	Brevibacterium linens	No classified. Linocina M18,	65	2.32E−13
BAC198	k95_707_349_716_−	Streptococcus equi	No classified. Zoocina A,	35.1	1.04E−10
BAC198	k95_6692_106_743_−			36.667	3.01E−07
BAC198	k95_7969_1_820_−			32.11	3.05E−06
BAC198	k95_6496_1_638_+			29.412	1.73E−05
BAC198	k95_10806_521_1761_+			30.851	4.67E−05

**TABLE A5:** Bacteriocins detected in Escarcega metagenome

Query acc.ver	Subject acc.ver	Taxonomic Group	Bacteriocin detected	% identity	e‐value
BAC133	k95_4234_1_553_	Lactobacillales	Clase III. Enterolisina	33.6	1.47E−05
BAC134	k95_15016_1_615_+	Lactobacillus helveticus	Clase II. Helveticina J,	47.1	6.15E−46
BAC137	k95_12217_237_1907_−	Brevibacterium linens	No clasified. Linocina M18	53.5	3.55E−90
BAC198	k95_212_1_516_+	Streptococcus equi	No clasified. Zoocin A	35	3.02E−12
BAC198	k95_6690_1_296_−			38.3	4.94E−08
BAC198	k95_4234_1_553_−			32.1	4.31E−06
BAC198	k95_9733_3153_3790_+			35.8	5.05E−06
BAC198	k95_1833_1_1932_−			30.5	2.35E−05
BAC198	k95_13060_1_371_−			29.7	2.57E−05
BAC198	k95_12773_129_1939_+			31.9	4.92E−04
